# SARS-CoV-2 in Nursing Homes after 3 Months of Serial, Facilitywide Point Prevalence Testing, Connecticut, USA

**DOI:** 10.3201/eid2705.204936

**Published:** 2021-05

**Authors:** Hanna Y. Ehrlich, Adora Harizaj, Lauren Campbell, McKenzie Colt, Karen Yuan, Therese Rabatsky-Ehr, Daniel M. Weinberger, Vivian Leung, Linda M. Niccolai, Sunil Parikh

**Affiliations:** Yale University, New Haven, Connecticut, USA (H.Y. Ehrlich, L. Campbell, M. Colt, K. Yuan, D.M. Weinberger, L.M. Niccolai, S. Parikh);; Connecticut Department of Public Health, Hartford, Connecticut, USA (A. Harizaj, T. Rabatsky-Ehr, V. Leung)

**Keywords:** COVID-19, coronavirus disease, SARS-CoV-2, severe acute respiratory syndrome coronavirus 2, viruses, respiratory infections, zoonoses, epidemiology, nursing home, outbreak, universal testing, RT-PCR, prevalence, long-term care facility, diagnostics, Connecticut, United States

## Abstract

Nursing homes house populations that are highly vulnerable to coronavirus disease. Point prevalence surveys (PPSs) provide information on the severe acute respiratory syndrome coronavirus 2 infection status of staff and residents in nursing homes and enable isolation of infectious persons to halt disease spread. We collected 16 weeks of public health surveillance data on a subset of nursing homes (34/212) in Connecticut, USA. We fit a Poisson regression model to evaluate the association between incidence and time since serial PPS onset, adjusting for decreasing community incidence and other factors. Nursing homes conducted a combined total of 205 PPSs in staff and 232 PPSs in residents. PPS was associated with 41%–80% reduction in incidence rate in nursing homes. Our findings provide support for the use of repeated PPSs in nursing home staff and residents, combined with strong infection prevention measures such as cohorting, in contributing to outbreak control.

Nursing home residents represent a population highly vulnerable to the spread of severe acute respiratory syndrome coronavirus 2 (SARS-CoV-2). In the midst of the coronavirus disease (COVID-19) pandemic, nursing homes account for a substantial proportion of total deaths attributed to the virus in the United States and globally (*1*–*3*). The high proportion of asymptomatic, presymptomatic, and atypical manifestations of COVID-19 in staff and elderly residents is a critical driver of widespread and rapid transmission of the virus (*4*–*6*). Facilitywide testing is a critical tool to identify such infections, particularly in lieu of effective vaccines or treatments early in a novel viral outbreak (*7*–*10*). Point prevalence surveys (PPSs) enable testing of populations at a specific point with the goal of isolating both infectious and exposed persons from unexposed, uninfected persons to prevent ongoing transmission.

Nursing homes in the state of Connecticut experienced a high burden of COVID-19 during the first surge of the pandemic. The first COVID-19 case was reported in a nursing home in Connecticut on March 15, 2020. Over the next 2 months, nursing homes accounted for 61.6% deaths in the state (*6*). After an increase in testing resources and evidence of asymptomatic transmission, the Connecticut Department of Public Health (CT DPH) began PPS testing in early May, and PPS testing was formally recommended on May 11 and mandated weekly in staff effective June 14 (*11*,*12*). Facility staff were trained by public health practitioners to ensure proper separation (hereafter, cohorting) of infected, exposed, and uninfected unexposed persons after receiving PPS results and temporary exclusion of staff from the workplace (*13*,*14*). Because data were collected for public health surveillance, not research, institutional review board evaluation was not required.

We previously reported the results of the first round of PPS testing in a subset of Connecticut nursing homes, in which a high number and proportion of asymptomatic infections were detected (*6*). We also discussed the rapid turnaround time from conduct of PPS and institution of cohorting in those initial PPSs, factors that probably contributed to the positive effect of PPSs in reducing transmission. In this observational study, we followed the same nursing homes as they conducted serial PPS testing. We describe 4 weeks of incidence data before initial PPSs and 12 weeks of follow-up data in which facilities underwent 1–11 additional PPSs. We also present the results of PPSs conducted in staff in the selected subset of nursing homes as well as from the first round of PPSs in nearly all (n = 196/212) nursing homes in the state. 

## Methods

### Nursing Home Selection

Due to limitations in testing resources at the start of PPS rollout, CT DPH prioritized specific nursing homes to receive test kits based on the size of their outbreaks and potential immediate effect of control measures. Of 212 nursing homes in the state, 34 conducted the first round of PPS testing on or before May 20, 2020, and were selected for extended follow-up in this study; 1 of these homes was COVID-19–naive and excluded from our previous study (*6*). The homes selected for inclusion in this study were of average size and quality of nursing homes in the state, with an average of 135 licensed beds and quality rating of 3.58/5 stars (*6*). By June 25, a total of 196 (92.5%) of 212 nursing homes throughout Connecticut had conducted >1 round of resident PPS testing and were included for reporting of initial results.

### PPS Testing, Cohorting, and Simultaneous Interventions

PPS involved molecular SARS-CoV-2 testing by nasopharyngeal swabs of all residents or staff in a facility within a short time period, in general 1 day (*6*). The state of Connecticut mandated weekly PPS testing in staff to begin in the latter half of June. In mid-May, CT DPH recommended but did not mandate weekly PPS testing of residents after identification of a new nursing home–onset case until no new cases were detected in residents or staff for 14 days (*11*,*12*). These recommendations remained effective through the duration of the study period. Nursing homes were paired with affiliate hospitals or laboratories to help conduct PPS testing and ensure fast turnaround of results.

A primary goal of PPSs was to ensure rapid and comprehensive isolation and cohorting of infected persons and to enact other infection prevention and control (IPC) measures, such as contact tracing to identify exposures and temporary exclusion of infected staff from the workplace. We did not collect data on adherence to these measures in nursing homes.

COVID-19 cases were also detected between PPSs, primarily through selective screening of residents leaving or entering the facility, visiting healthcare settings, or experiencing relevant symptoms, and also through limited contact tracing. Many other IPC policies for nursing homes were enacted during the study period federally and in the state of Connecticut, which can be found in Appendix C of the CT DPH contracted report by Mathematica, Inc. (*15*).

### Data Extraction

Nursing home staff answered daily questionnaires in a web-based COVID-19 database maintained by CT DPH, through which we extracted data on daily case counts, deaths, and censuses. PPS results were confirmed with study investigators by telephone: nursing directors reported the results of tests given to residents or staff who did not have a prior diagnosis of COVID-19. Case dates correspond to the date of specimen collection. We were unable to follow up on how each lab and nursing home responded to inconclusive results: whether they repeated the test, acquired a new sample, or treated the result as positive. New cases excluded residents transferred in with a known SARS-CoV-2 infection. Case counts by town were obtained from the Connecticut COVID-19 portal (*16*). 

### Incidence Rates Relative to First PPS

**Figure 5:**
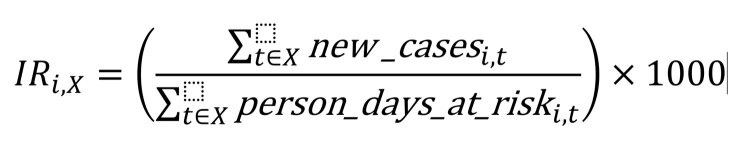
Equation for each respective time period *X* and nursing home .

COVID-19 incidence rates were calculated for 3 time periods respective to each nursing home: 4 weeks before first PPS, day of first PPS (“day 0”), and 12 weeks after the first PPS. For each respective time period *X* and nursing home , we used the following equation: Figure 5where person-days at risk on day *t* was calculated as the resident census reported on day *t,* subtracting the number of previous COVID-19 case-patients who had not died from complications of the disease by day *t.* The total number of cases and person-days at risk in nursing home *i* was summed for all days within each time period *X*. Because we could not follow individual persons over time, we used census data to account for the dynamic nature of nursing home populations. We compared the incidence rates in PPS in individual nursing homes in the 4 weeks prior and 12 weeks following first using the 2-sample Z-test for equality of proportions with Yates’ continuity correction.

### Poisson Regression Model

**Figure 6:**
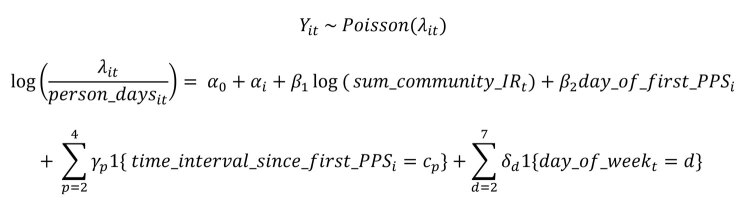
Equation for the number of new cases in nursing home on calendar day offset by person-days at risk on day *t* was modeled as a Poisson regression.

We investigated the association between PPS and the trajectory of nursing home outbreaks while accounting for concomitant changes in community incidence and intrinsic variability between nursing homes. The number of new cases in nursing home on calendar day offset by person-days at risk on day *t* was modeled as a Poisson regression: Figure 6where *person_days_it_* is as described previously; *sum_community_IR_it_* is the incidence rate per 100,000 population in the town in which nursing home *i* is located over the past 14 days relative to day *t* (*17*); *day_of_first_PPS_i_* is the date of the first PPS, included as a dummy variable to account for the substantial change in screening practices; and *time_interval_since_first_PPS_i_*is treated as a categorical variable divided into 1–15, 16–30, 31–60, and 60–90 days. Categorical variables for day of the week and nursing home ID (*α_i_* were also included. The model did not exhibit evidence of overdispersion (deviance/degrees of freedom = 0.8), indicating that the Poisson model was appropriate. We conducted a sensitivity analysis to determine the impact of different lags of community incidence (0, 3, 7, 14, and 28 days) on model results; the sum of incidence over the previous 14 days was found to minimize the Akaike information criterion. Risk ratios were calculated by exponentiation of the relevant regression coefficients. Analyses and figures were executed in R version 3.5.1 (https://www.r-project.org).

## Results

### PPS Implementation

In the 12 weeks of follow-up after initial PPSs, an average of 6.0 (range 1–10) follow-up PPSs in residents and 6.2 (range 2–10) total PPSs in staff were administered per nursing home, for a total of 198 follow-up surveys in residents and 205 surveys in staff in all 34 nursing homes (Table 1). The average time between the first and second round of resident PPS testing was 30 days; average time between all subsequent PPSs was 9 days. Periods between staff PPSs were shorter than between resident PPSs (Appendix Figure 1). The period between resident PPSs decreased over time, in part, because of additional state requirements and recommendations to conduct weekly resident testing in mid-July. Most (31/34) nursing homes in this study conducted >1 PPS beyond the recommended threshold of 14 days after a positive case was detected. The total number of PPSs in residents and staff in each nursing home was not statistically associated with the nursing home quality rating.

**Table 1:** Summary of point prevalence survey results of severe acute respiratory syndrome coronavirus 2 infection in 34 nursing homes, Connecticut, USA*

Category	Residents						Staff	
	No. follow-up PPS	Positive test results from PPSs†	No. symptomatic at PPS testing	No. cases detected between PPSs†	No. symptomatic at time of non-PPS testing		No. PPS	Positive test results from staff PPSs
Total	198	44	11	93	70		205	87
Average (SD)	6.0 (2.3)	1.3 (1.5)	0.6 (0.9)	2.7 (7.6)	4.1 (9.1)		6.2 (2.0)	2.6 (4.9)

*Results of the first PPS in residents in (6); results displayed here are those of subsequent surveys only. In brief, 601 cases were detected in the first PPS (average 16.8, SD 13.5). One additional facility, coronavirus disease naive at the time of the initial PPS and therefore not included in the original study, detected 0 cases in its first PPS. PPS, point prevalence survey. †Excludes residents transferred into facilities with known coronavirus infection.

### Resident Cases Detected in Follow-Up Period

Before the first PPS, nursing homes had experienced an average of 36 COVID-19 cases (27.7% infected; range 0–81 cases, 0%–86.1% infected). A total of 601 cases were detected in these facilities during the first PPS, as previously described (*6*). Approximately 1,775 (55.8%) of all residents in the study were assumed to be susceptible to infection after the first round of testing was complete.

**Figure 1:**
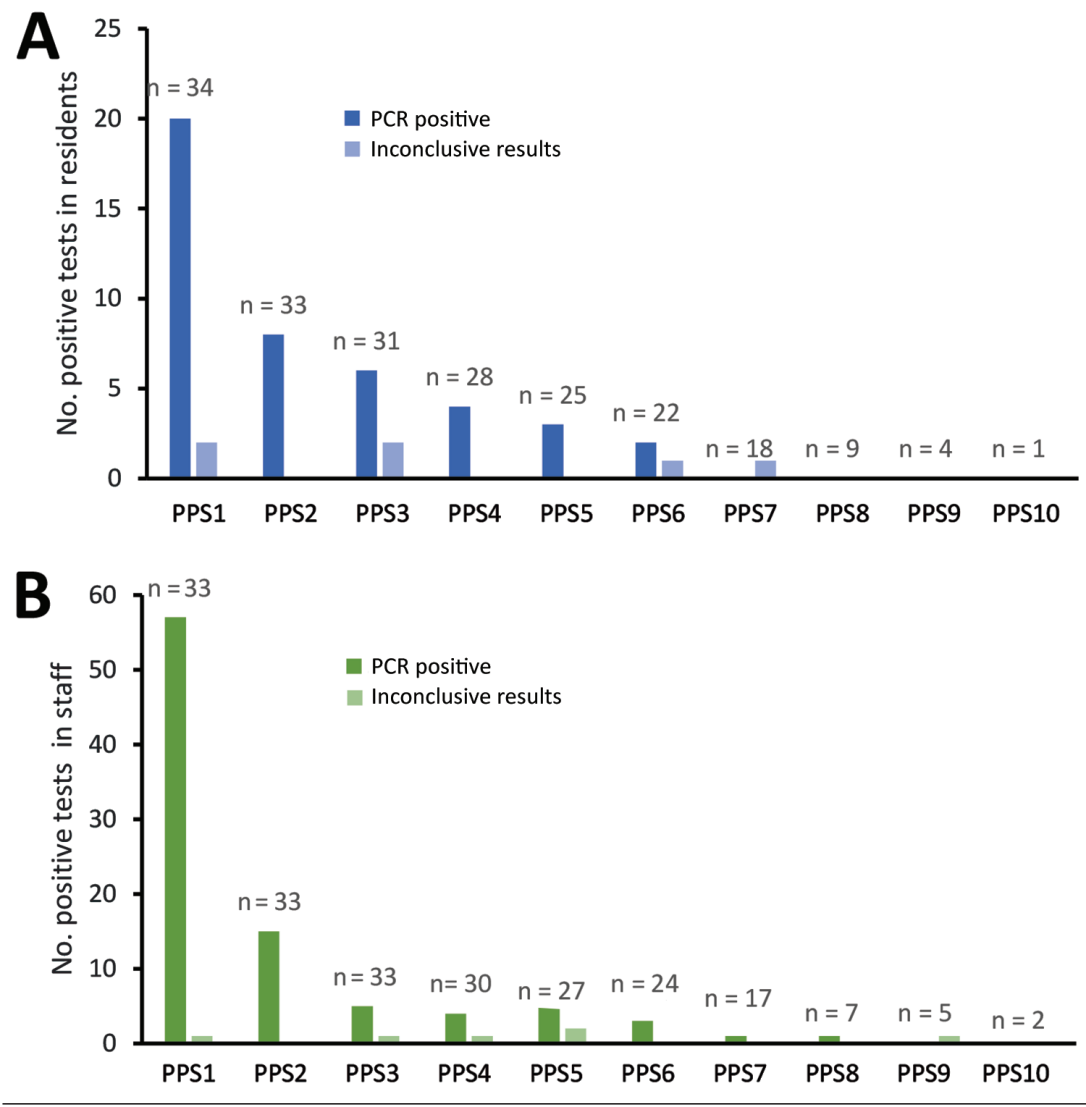
Coronavirus disease cases detected in consecutive PPSs in residents (A) and staff (B) in nursing homes, Connecticut, USA. The number of participating nursing homes for each survey is listed above each bar. One facility was excluded from staff testing data due to lack of verifiable testing results during PPS surveys. The results of the first PPS in residents, in which 601 cases were detected, were previously reported in (*6*). The probability of detecting a positive case decreased significantly (p<0.05) through PPS7 for residents and PPS8 for staff, compared with the first PPS, using logistic regression for comparisons. PPS, point prevalence survey.

After the initial round of PPS, a total of 44 resident cases were identified in all subsequent rounds of PPS testing, of which 9 (20.4%) were symptomatic at the time of testing (Table 1). The probability of identifying additional cases through PPSs decreased significantly over subsequent PPSs: the second PPS identified 20 cases (n = 34 nursing homes), and subsequent PPSs identified an additional 8 (n = 33), 6 (n = 31), 4 (n = 28), 3 (n = 25), 2 (n = 22), 0 (n = 18), 0 (n = 9), 0 (n = 4), and 0 (n = 1) cases in residents (Figure 1).

**Figure 2:**
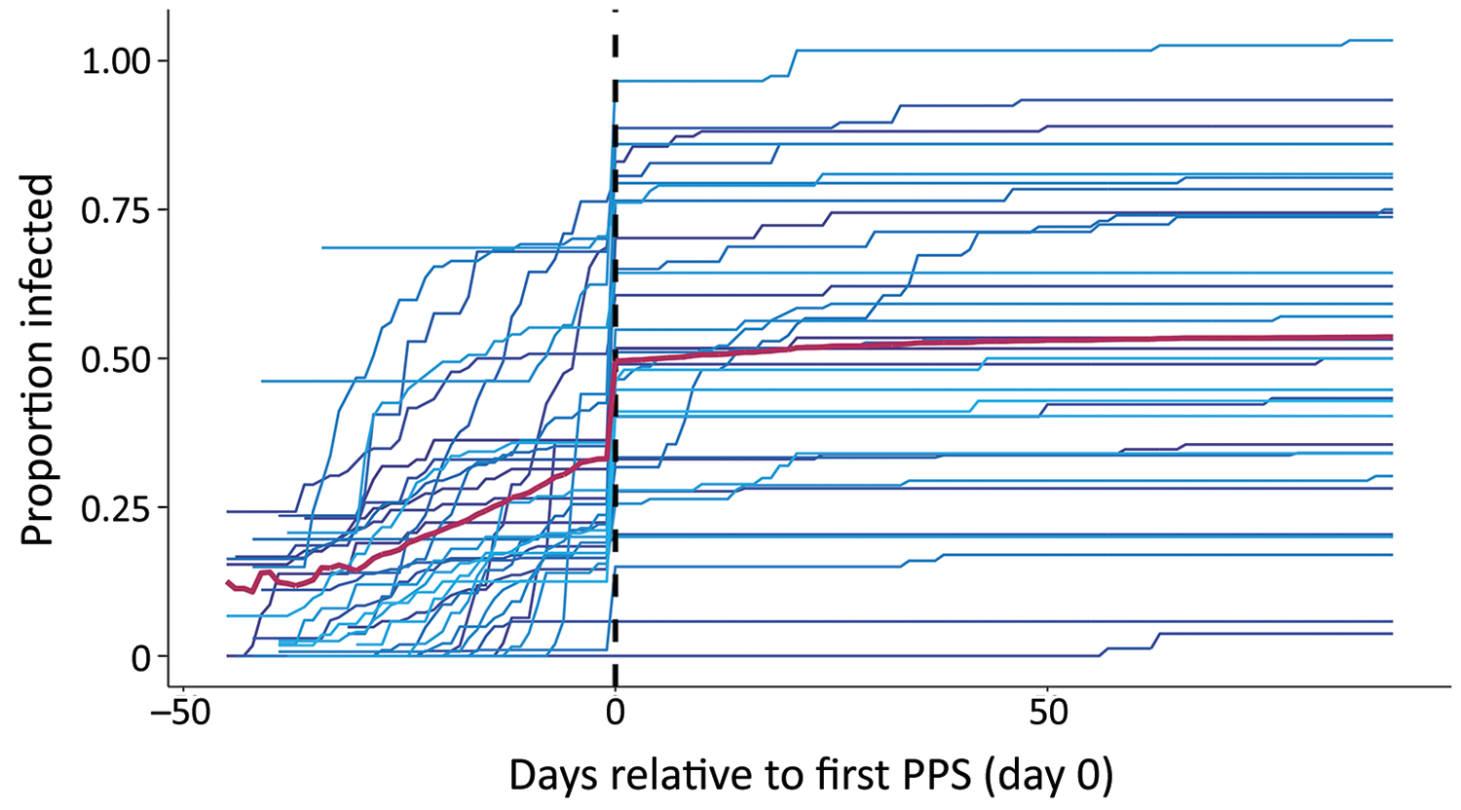
Cumulative proportion of severe acute respiratory syndrome coronavirus 2 (SARS-CoV-2) infections in individual nursing homes over a 16-week study period relative to the first PPS, Connecticut, USA. Each colored line represents a single nursing home in the ≈4 weeks before first PPS and 12 weeks following first PPS. Data were centered for all nursing homes by the date of receipt of results for the first PPS, signified by the dashed vertical line on day 0. Red line indicates average proportion infected of the total study population on each day. The number of residents infected in each nursing home is based on cumulative case counts out of the number ever susceptible to SARS-CoV-2 in the nursing home, or the maximum census value in the study period, to account for resident deaths and transfers since the start of reporting. PPS, point prevalence survey.

In between PPSs, 93 additional resident cases were also detected, of which 70 (75.3%) were symptomatic at the time of testing. Most (85, 90.3%) cases were identified during the longer period between the first and second round of PPS testing. More than half (60.2%) of cases were detected within 1 incubation period following the first PPS, when exposure in those persons had likely already occurred; that exposure rendered cohorting measures less effective. Further, there was a positive but nonsignificant correlation (p = 0.09) between the number of days between PPS and the number of cases identified in a nursing home (Appendix Figure 2). Two nursing homes contained most of these cases, reporting 38 and 20 cases in the 44 days between their first and second PPS (Figure 2; Appendix Figures 2, 3).

### Temporal Patterns of Resident Infections

Nursing homes underwent initial PPS at different stages of outbreak severity (Figure 2). After initial PPS, the proportion of residents infected in each nursing home plateaued for most facilities. In 41.2% of nursing homes, fewer than half of all residents were infected with SARS-CoV-2 by the end of the study period.

**Figure 3:**
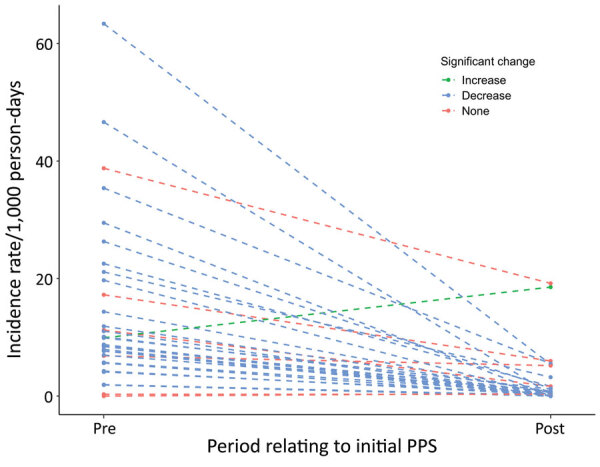
Paired coronavirus disease incidence rate estimates relative to first PPS, Connecticut, USA. Dashed lines represent single nursing homes included in the study. Points represent the incidence in the 4 weeks before the first PPS and 12 weeks following the first PPS, during which additional PPSs were also conducted. Blue indicates significant decreases in incidence for each nursing home over the 2 time periods (α = 0.05); green indicates significant increases; red indicates nonsignificant changes in incidence. PPS, point prevalence survey.

The median incidence rates in nursing homes were 9.3 (95% CI 0.2–49.2) cases/1,000 at-risk person-days before the first PPS; 267.8 (95% CI 0–861.5) cases/1,000 person-days on the day of the first PPS, and 0.54 (95% CI 0–18.4) cases/1,000 person-days in the period after the first PPS. Incidence rates decreased (p<0.05) in 85% (29/34) of facilities following the implementation of PPSs (Figure 2). Of the 4 nursing homes that experienced no significant change, 2 had <10 residents remaining susceptible to SARS-CoV-2 and 1 had not experienced any cases before the first PPS. Meanwhile, 2 nursing homes experienced large outbreaks of >10 cases after the first PPS, 1 of which experienced an increase in incidence rate of 8.3 cases/1,000 person-days (Figure 3; Appendix Figure 3).

### Accounting for Concurrent Changes in Community Incidence

**Figure 4:**
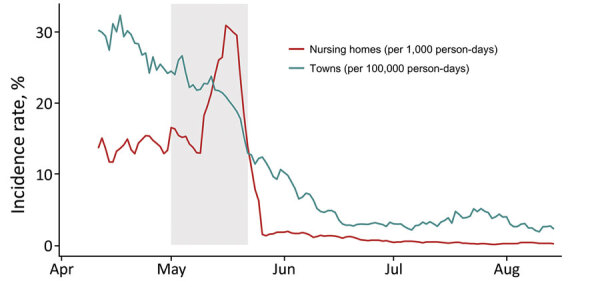
Coronavirus disease incidence rates in nursing homes (cases/1,000 person-days, red) and in towns and cities (cases/100,000 person-days, blue), Connecticut, USA. Incidence rates are aggregated for the 34 nursing homes in this study and 26 towns and cities in which the nursing homes are located; incidence is presented as rolling weekly averages to account for differences in day-of-week reporting. The shaded rectangle shows the time period in which all 34 nursing homes conducted initial PPSs.

The population of the towns and cities in which the nursing homes were located experienced a contemporaneous decrease in community incidence during the study period (Figure 4). Community incidence over the previous 2 weeks was associated with proportional changes in incidence in nursing homes (β_1_ = 0.98, 95% CI 0.84–1.11). After adjusting for community incidence and the change in screening practices, the implementation of serial PPSs was associated with a significant decrease in nursing home incidence rates of 77% (95% CI 71%–83%) in the first 15 days after the first PPS, 49% (95% CI 31%–63%) from days 16–30, 41% (95% CI 12%–60%) from days 31–60, and 80% (95% CI 64%–89%) reduction from days 61–90, compared with the pre-PPS period.

### Staff Cases Detected in Follow-Up Period

Nursing homes identified 87 staff cases (6 inconclusive) or an average of 2.6 cases (SD 4.9) per facility in the follow-up period (Table 1). The first PPS in 34 nursing homes identified 57 total staff cases, and subsequent PPSs (n = 33 nursing homes) identified an additional 15 (n = 34 nursing home’s staff tested), 5 (n = 33), 4 (n = 30), 5 (n = 27), 3 (n = 24), 1 (n = 17), 1 (n = 7), 0 (n = 5), and 0 (n = 2) staff cases (Figure 1). Symptomatic status and cases counts identified outside of weekly PPSs were not ascertained. One nursing home was removed from staff testing results beyond the first PPS due to lack of verifiable data.

### Statewide Initial PPS Testing

In the state of Connecticut, as of June 25, 2020, a total of 196 nursing homes had completed 1 round of PPS testing. In these initial single round of surveys, 12,336 residents were tested. A total of 1,733 tests (14.0%) were SARS-CoV-2 positive and an additional 70 tests were inconclusive. Of those with positive results, 1,537 (88.7%) were reported by facilities as having been asymptomatic at the time of testing. Follow-up for symptomatic status beyond the day of testing was not conducted.

## Discussion

We compiled a large dataset covering 16 weeks of public health surveillance data in nursing homes, documenting COVID-19 outbreaks in the 4 weeks before and 12 weeks after the start of repeated facility-wide PPSs. Several previous studies have also documented the successful implementation of PPS testing in multiple congregate living facilities in the context of COVID-19 outbreak control (*4*,*7*,*8*,*18*–*26*). We describe a study of 34 facilities conducting 437 surveys in residents and staff and 35,133 nasopharyngeal swab tests, or an average of 13 PPSs per nursing home in residents and staff combined in a 12-week period. Selected nursing homes experienced a range of outbreak severities at the time of initial PPSs, yet all nursing homes experienced 1 or 0 cases in the final 4 weeks of follow-up. In addition, 29/34 (85%) nursing homes exhibited significant (p<0.05) decreases in incidence rates of SARS-CoV-2 infection in the 12-week follow-up period compared with the 4-week period before any PPS.

The initial round of PPS testing likely captured asymptomatic cases and residents with protracted viral shedding that had been missed in the pre-PPS period (and who may have been symptomatic at that time), as well as presymptomatic cases that would have been captured in the post-PPS period in lieu of PPSs (*6*,*27*,*28*). To account for the change in screening practices, we compared trends in incidence rates before and after initial PPSs. The change in incidence rates of COVID-19 cases in nursing homes over the study period, especially in the period following the first round of PPS, coincided with a decrease in community cases. However, we found that, even after adjusting for community incidence and the change in screening practice, the decrease in incidence rates in nursing homes was significantly associated with the onset of PPSs (p<0.05 for all subsequent time divisions).

Most COVID-19 cases detected in the 12-week follow-up period were identified in the extended period, on average 30 days, between the first and second PPS. These cases were identified primarily through symptom screening; limited contact tracing; and other types of selective testing, including at the time of resident hospitalization or hemodialysis. We postulate that more frequent PPSs, especially between the first and second rounds of testing, may have improved outbreak control by enabling earlier cohorting and that the extended time period between PPS may have decreased the efficacy of this intervention overall. Our results also suggest that although introductions of the virus from staff, visitors, and patients undergoing outside procedures pose a substantial risk of seeding new outbreaks, nursing homes may be able to alter the trajectory of their outbreaks by rigorous case surveillance once an outbreak occurs, despite ongoing community transmission.

Our study’s limitations include that we were not able to incorporate a control group in this analysis because this work was done in the context of outbreak control, in which nearly all nursing homes in the state received PPSs over the follow-up period. Furthermore, we were not able to follow individual participants over time due to the dynamic nature of nursing home populations and limitations in public health surveillance capacity. Similarly, we were unable to collect data to track the implementation of cohorting and other behavioral and physical interventions after receiving test results from PPSs. Nonetheless, CT DPH staff called all facilities before the first PPS to assess knowledge of cohorting and train staff on appropriate cohorting; they also followed up on homes with continued transmission after the first PPS to evaluate adherence. Finally, we could not account for all concurrent interventions, including changes in visitation policies, staff cohorting practices, and PPE abundance, limiting the interpretability of the usefulness of repeated PPSs (*15*).

We described the successful implementation of hundreds of repeated facilitywide PPSs in nursing homes. Although our findings cannot inform policies of asymptomatic testing of staff and residents as a preventive strategy, they suggest that PPSs is one of several effective tools in outbreak management, particularly in the context of low COVID-19 incidence in the general population. In addition to testing, outbreak control relied on use of PPE and other protective behaviors such as social distancing and limitations to visitation, successful cohorting of infected and exposed residents, exclusion of infected staff from the workplace, environmental modifications, and sustained IPC training (*18*). Our work may motivate states to reserve financial resources for sustained, serial PPS testing in the context of outbreak control and other forms of IPC planning in long-term care. We urge policymakers to continue serial testing in congregate living facilities during the period of vaccine rollout because acquisition of immunity will take time and coverage rates may vary in facilities (*9*,*29*,*30*). Optimal serial testing strategies in the post–vaccine rollout period will require additional study.

Appendix Additional information about severe acute respiratory syndrome coronavirus 2 in nursing homes after 3 months of serial point prevalence testing, Connecticut, USA.
