# Supplementary material for: SARS-CoV-2 in Nursing Homes after 3 Months of Serial, Facilitywide Point Prevalence Testing, Connecticut, USA
Source: Emerg Infect Dis. 2021 May;27(5):1288–95. doi: 10.3201/eid2705.204936 (PMC8084507; doi:10.3201/eid2705.204936)
Supplement: Appendix — Additional information about severe acute respiratory syndrome coronavirus 2 in nursing homes after 3 months of serial point prevalence testing, Connecticut, USA. [file 20-4936-Techapp-s1.pdf]

Article DOI: https://doi.org/10.3201/eid2705.204936

SARS-CoV-2 in Nursing Homes after 3 Months of Serial, Facilitywide Point Prevalence Testing, Connecticut, USA

Appendix

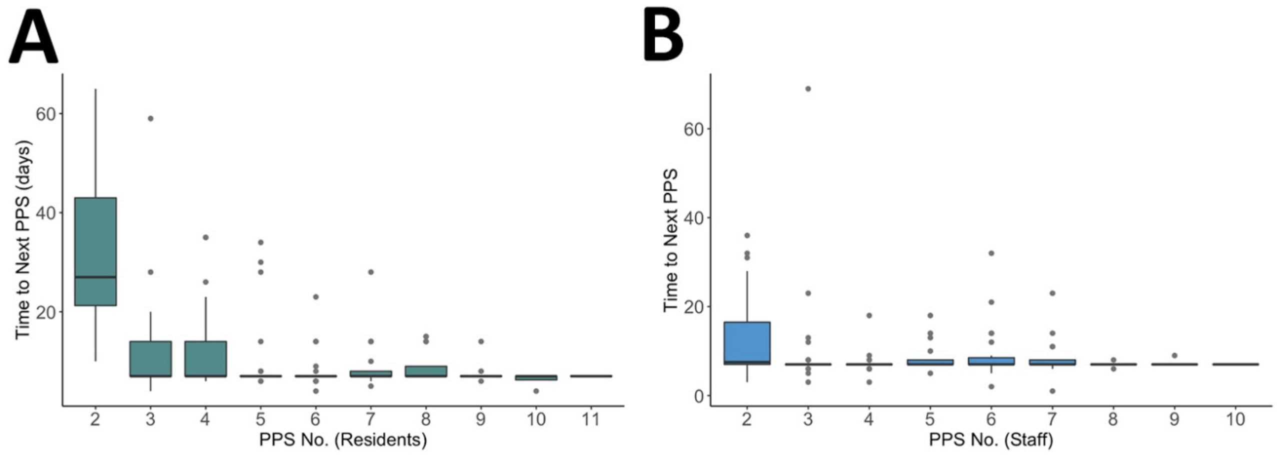

Appendix Figure 1. Time in days to subsequent point prevalence survey (PPS). Horizontal line in each box indicates median number of days (horizontal line) from the first PPS to the second PPS, and so forth, for residents (left) and staff (right). Top and bottom borders indicate 75% interquartile range (IQR), whiskers represent the range of values 1.5 times the IQR, and dots represent outliers The probability of detecting a positive case in subsequent PPSs were compared with the reference (initial) PPS using logistic regression.

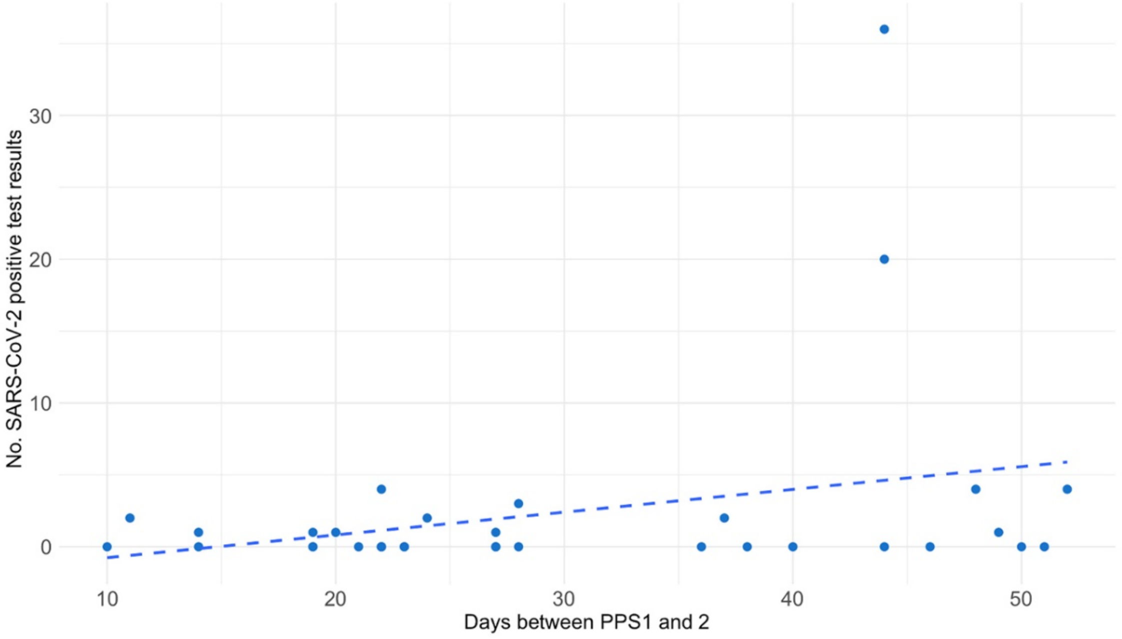

Appendix Figure 2. Cases detected between first and second point prevalence survey (PPS). Association between the number of days from the first to the second PPS and the respective total cases (i.e., number of persons with positive SARS-CoV-2 test results) identified in the same time period ($R^2 = 0.08$, $p = 0.09$). Each dot represents a single nursing home. Dashed line represents the line of best fit.

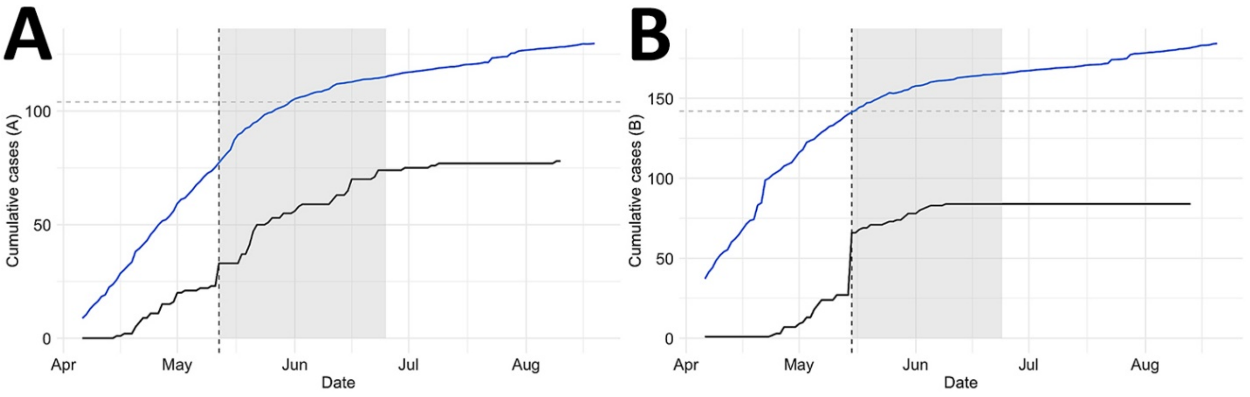

Appendix Figure 3. Outbreaks of coronavirus disease in 2 nursing homes after first point prevalence survey (PPS). Two nursing homes (A, left and B, right) experienced outbreaks after initial PPSs, defined here as detecting the virus in >10 residents at any point after the first PPS. The plots show the cumulative number of nursing home cases (black lines) and total cases of the county in which the nursing home was located (blue lines) divided by 100 for scale. The gray rectangles represent the time between the first and second PPS; vertical dashed lines represent the date of the first PPS; horizontal dashed lines represent the number of persons ever susceptible to SARS-CoV-2 in the respective nursing home.
